# Effectiveness of anticoagulant therapy using heparin combined with Plavix after Rex shunt

**DOI:** 10.3389/fped.2024.1339348

**Published:** 2024-02-05

**Authors:** JinShan Zhang, Long Li

**Affiliations:** ^1^ Department of General Surgery, Capital Institute of Pediatrics, Beijing, China; ^2^ Research Unit of Minimally Invasive Pediatric Surgery on Diagnosis and Treatment, Chinese Academy of Medical Sciences, Beijing, China

**Keywords:** extra-hepatic portal venous obstruction, children, Rex shunt, anticoagulant therapy, heparin

## Abstract

**Purpose:**

Rex shunt is an optimal surgery for the treatment of extra-hepatic portal venous obstruction (EHPVO) in children. Anticoagulant therapy has been used to keep the patency of the bypass vein in the Rex shunt. This study was to investigate the effectiveness of anticoagulant therapy using heparin combined with Plavix in improving the prognosis and shunt patency of Rex shunt.

**Methods:**

From January 2010 to September 2019, 51 children with EHPVO underwent a portal cavernoma- Rex shunt. Based on whether using the anticoagulant therapy after the Rex shunt, all patients were divided into two groups: the anticoagulant group and the non-anticoagulant group. The diameter and flow velocity of the bypass vein were measured by the post-operative ultrasound, which was used to calculate the flow volume of the bypass vein (FV) and standard portal venous flow (SPVF). The bypass venous flow index (BVFI) was used to evaluate the ability of portal blood into the liver through the bypass vein after the Rex shunt, which was a ratio of FV to SPVF. The incidence of post-operative re-bleeding, the postoperative patency rate of the bypass vein, the remission rate of postoperative hypersplenism, the remission rate of postoperative esophagogastric varices and the BVFI were compared between the two groups.

**Results:**

Of the 51 patients, 12 patients in the anticoagulant group were treated with heparin combined with Plavix after Rex shunt; 39 patients in the non-anticoagulant group were not treated with any anticoagulant therapy. 8 of 51 patients suffered from postoperative re-bleeding, of whom 6 patients with thrombosis of the bypass vein and 2 patients with anastomotic stenosis of the bypass vein. All 8 patients with re-bleeding belonged to the non-anticoagulant group. The remission rate of hypersplenism was no significant difference between the two groups after surgery (91% vs. 58%, *P* = 0.100). However, 3 patients without hypersplenism before surgery suffered from hypersplenism after surgery, who belonged to the non-anticoagulant group. There was no significant difference in the remission rate of esophagogastric varices (33% vs. 46%, *P* = 1.000). The BVFI of the anticoagulant group was significantly higher than that of the non-anticoagulant group (5.71 ± 5.89 vs. 1.1 ± 1.52, *P* = 0.003).

**Conclusions:**

Anticoagulant therapy using heparin combined with Plavix plays an important role in maintaining the patency of the bypass vein, which improved the portal blood flow into the liver through the bypass vein after the Rex shunt.

## Introduction

Rex shunt could relieve portal hypertension and restore the portal blood into the liver effectively in extra-hepatic portal venous obstruction (EHPVO) by reconstructing the passageway that portal blood into the liver. Rex shunt was considered as the most ideal surgical method in the treatment of EHPVO in children (1–3). However, the failure rate of the Rex shunt was 8%–40% (4–9), which was usually caused by thrombosis or stenosis of the bypass vein (10). Anticoagulant therapy is supposed to be a valuable therapy for avoiding thrombosis of the bypass vein in the Rex shunt. Various postoperative anti-coagulation strategies have been used to prevent shunt thrombosis, but reported protocols vary widely among groups (7, 11–19). However, we didn't use the anticoagulant therapy after Rex shunt before 2018 due to the following reasons: First, the anticoagulant therapy might increase the risk of post-operative re-bleeding; Second, we believed the anti-coagulant therapy was not an important factor in keeping the patency of the bypass vein. However, the failure rate after Rex shunt was 19% before 2018(3). In 2018, we found that post-operative anticoagulant therapy was routine management in most centers after Rex shunt. Since then, we began to use anticoagulant therapy after Rex shunt to reduce the failure rate after Rex shunt. Since 2018, post-operative anticoagulant management has been used after Rex shunt in our cases: following surgery, children received an intravenous infusion of heparin for 7 days followed by oral Plavix for 6 months. This study was performed to evaluate the effectiveness of this post-operative anticoagulant regimen by comparing the prognosis of patients with and without postoperative anticoagulant therapy after Rex shunt.

## Materials and methods

From January 2010 to September 2019, 51 children (32 males and 19 females, age at operation: 2.1 to 17.8 years, median age: 5.3 years) suffered from upper gastrointestinal bleeding because of EHPVO. All patients were treated with a Rex shunt (Portal cavernoma -Rex shunt with interposition of grafted portal vessel). A single surgeon operated on all patients in the study population, and the operative technique has been described previously (20), in which the inferior mesenteric vein was used as the grafted bypass vein. There was no other underlying diseases in all patients. The levels of anti-coagulant factors including protein C (PC), protein S (PS) and anti-thrombin III (ATIII), routine blood test, liver function, blood ammonia and coagulant function before and after Rex shunt were detected. Because the intraoperative portal venography and liver biopsy were usually performed in our cases, we didn't perform the preoperative wedge portographies and liver biopsies to identify ideal patients for Rex shunt. All patients had no previous history of a portosystemic shunt or Rex shunt.

Since 2018, we have started to perform anticoagulant therapy after the Rex shunt. The anticoagulant regimen includes the continuous intravenous infusion of heparin (10 U/kg/h) was performed for 7 days after surgery, and then oral Plavix (1 mg/kg/d) was used for 6 months after discharge. The patients treated with this anticoagulant regimen are regarded as the anticoagulant group, and those without any anticoagulant therapy including anti-platelet therapy are regarded as the non-anticoagulant group. The postoperative activated partial thrombin time (APTT) was monitored after surgery in all patients.

### Follow-up

All the children were followed up at postoperative 1, 3, 6, and 12 months, and every 6 months thereafter. During the follow-up, abdominal ultrasonographic studies (US), abdominal computed tomography (CT), upper gastrointestinal radiography (UGI), and laboratory tests (routine blood test, liver function, coagulation function, and blood ammonia) were conducted at each visit.

### Evaluation of the prognosis

**Figure 1:**
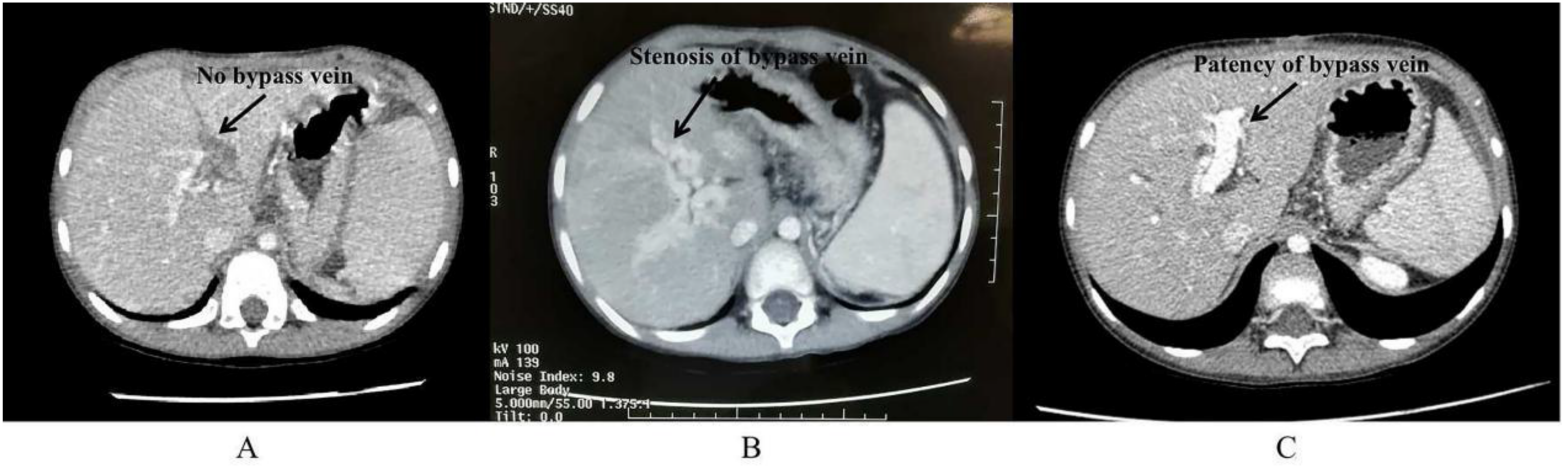
The CT after Rex shunt. The disappearance of the bypass vein is shown by CT (**A**); the stenosis of the bypass vein is shown by CT (**B**); the patency of the bypass vein is shown by CT (**C**).

1. The postoperative re-bleeding: The hematemesis or melena during following-up was defined as the postoperative recurrence of upper gastrointestinal bleeding. The incidence of postoperative re-bleeding was compared between the anticoagulant group and the non-anticoagulant group.
2. The patency of the bypass vein: If the bypass vein cannot be detected by US and CT, the thrombosis of the bypass vein was diagnosed (Figure 1A). Generally, the diagnosis of shunt stenosis is made when the diameter of the bypass vein is less than 3 mm in US or CT (21, 22). Therefore, the diameter of the bypass vein with less than 3 mm shown by the US was considered as the stenosis of the bypass vein in this study (Figure 1B). The thrombosis and stenosis of the bypass were regarded as no-patency of the bypass vein. The postoperative patency rate of the bypass vein was compared between the anticoagulant group and the non-anticoagulant group.
3. The hypersplenism: The hypersplenism was evaluated by the level of platelets. If the level of platelets is less than 100 × 10^9^/L, and splenomegaly is shown by US or CT, it is defined as hypersplenism. If the level of platelets returns to normal after the operation (more than 100 × 10^9^/L), and the splenomegaly is relieved shown by US or CT, it is defined as the postoperative remission of hypersplenism. The postoperative remission rate of hypersplenism was compared between the two groups.
4. The esophagogastric varices: If the esophagogastric varices was relieved shown by UGI, the postoperative remission of esophagogastric varices is defined. The postoperative remission rate of esophagogastric varices was compared between the two groups.
5. The bypass vein flow index (BVFI): The diameter (D) and maximum blood flow velocity (Vmax) of the bypass vein were measured by the US. The flow volume (FV) of the bypass vein was calculated by the formula [FV (ml/min) = *k* (0.57) × *V*_max_(cm/s) × 1/4 × 3.14 × 60(s) × D^2^ (cm^2^)]. The standard portal venous flow (SPVF) was calculated by the formula [SPFV (ml/min) = 30.1 × Age (years) −1.06 × Height (cm) + 3.31 × Weight (kg)] (23). The bypass vein flow index (BVFI) was a ratio of FV to SPVF, which was used to evaluate the ability of portal blood flow into the liver through the bypass vein after the Rex shunt. The BVFI was compared between the two groups.

### Statistical analysis

SPSS 13.0 statistical software (SPSS Inc., Chicago, IL, USA) was used to perform statistical analysis. The chi-square test and independent t-test were used for statistical analysis. *P*-value < 0.05 was considered statistically significant.

### Ethical statement

All procedures performed in studies involving human participants were under the ethical standards of the institutional research committee, the 1964 Helsinki declaration and its later amendments or comparable ethical standards. This work has been approved by the ethical committee of the Capital Institute of Pediatrics. All patients and their parents gave their informed consent before their inclusion in the study.

## Results

Of the 51 patients, 12 cases were in the anticoagulant group and 39 cases were in the non-anticoagulant group. There was no significant difference between the two groups in the ratio of gender, age at operation, incidence of preoperative esophagogastric varices, the incidence of preoperative hypersplenism, and splenic size before surgery (Table 1). All children were followed up after Rex shunt. The children in the anticoagulant group were followed up for 1–36 months with a mean of 7.8 months. The children in the non-anticoagulant group were followed up for 1–86 months with a mean of 18.6 months. However, there was no significant difference between the anticoagulant group and the non-anticoagulant group in the duration of follow-up (*P *= 0.135). The detailed results of the prognosis was shown in Table 2. 8 of 51 patients suffered from postoperative re-bleeding (haematemesis or/and melena) caused by varices, whom belonged to the non-anticoagulant group. The first time of re-bleeding was the post-operative 1–24 months with a mean of 12 months. Of the 8 patient with the postoperative re-bleeding in the non-anticoagulant group, 6 patients suffered from the thrombosis of bypass vein at the postoperative 18, 14, 12, 1.5, 1 and 1 months, respectively (Figure 1A), and 2 patients with the stenosis of bypass vein (Figure 1B). The mean time of thrombosis of the bypass vein in the no-anticoagulant group was 7.9 months. Of 2 patients with the stenosis of bypass vein, one patient underwent a re-Rex shunt after a failure of the interventional balloon dilatation, and the other patient underwent an interventional balloon dilatation and had a good outcome. The bypass vein was patent, and there was no thrombosis or stenosis in the remaining 43 patients shown by US and CT (Figure 1C). Because all patients had the patent bypass vein in the anticoagulant group, none patients with thrombosis still under plavix therapy.

**Table 1:** The general information of anticoagulant and non-anticoagulant groups before surgery.

Groups		Anticoagulant	No-anticoagulant	*P*
*n*		12	39	
Sex (male: female)		8: 4	24: 15	1.000
Age at operation (years)		7.0 ± 4.3	6.3 ± 2.9	0.850
Incidence of esophagogastric varices % (*n*)		50 (3/6)	63 (19 / 30)	0.658
Incidence of hypersplenism % (*n*)		92 (11 / 12)	58 (19 / 33)	0.074
Splenic size (cm)^a^	Length	14.8 ± 2.5	14.1 ± 2.8	0.470
	Thickness	4.5 ± 0.9	4.4 ± 1.1	0.744

^a^ The splenic size was measured by ultrasound. The information of patients was acquired before Rex shunt after admission.

**Table 2:** The difference of prognosis between anticoagulant and non-anticoagulant groups after surgery.

Groups		Anticoagulant	No-anticoagulant	*P*
*N*		12	39	
Incidence of post-operative re-bleeding % (*n*)		0 (0/12)	20.5 (8/39)	0.173
Patency rate of bypass vein % (*n*)		100 (12/12)	79.5 (31/39)	0.173
Remission rate of hypersplenism % (*n*)		91 (10/11)	58 (11/19)	0.100
Remission rate of esophagogastric varices % (*n*)		33 (1/3)	46 (5/11)	1.000
Duration of follow-up (months)		7.8 ± 11.6	18.6 ± 23.9	0.135
BVFI		5.71 ± 5.89	1.1 ± 1.52	
Bypass vein^a^	Diameter (mm)	8.1 ± 3.1	7.0 ± 2.4	0.358
	Flow velocity (cm/s)	43.9 ± 25.6	35.8 ± 28.4	0.125

^a^ The diameter and flow velocity of bypass vein were monitored by ultrasound. BVFI is the ratio of the flow volume of bypass vein (FV) to the standard portal venous flow (SPVF), which is an index evaluating the ability of portal blood into the liver through bypass vein. The information of patients was acquired after Rex shunt during the follow-up.

The hypersplenism was relieved after Rex shunt in 21 of 30 patients, in whom 10 of 11 cases belonged to the anticoagulant group (the postoperative remission rate of hypersplenism was 91%), and 11 of 19 patients belonged to the non-anticoagulant group (the postoperative remission rate of hypersplenism was 58%). There was no significant difference between the two groups in the postoperative remission rate of hypersplenism (*P *= 0.100). However, 3 patients without the preoperative hypersplenism suffered from the postoperative hypersplenism, who belonged to the non-anticoagulant group, of whom 2 children suffered from the postoperative re-bleeding caused by the thrombosis of bypass vein, and one patient with a patent bypass vein.

The esophagogastric varices were relieved in 6 of 14 patients after Rex shunt, in whom 1 of 3 patients in the anticoagulant group (the postoperative remission rate of esophagogastric varices was 33%), and 5 of 11 patients in the non-anticoagulant group (the postoperative remission rate of esophagogastric varices was 46%). There was no significant difference in the postoperative remission rate of esophagogastric varices between the two groups (*P *= 1.000).

The BVFI of the anticoagulant group was significantly higher than that of the non-anticoagulant group (*P *= 0.003).

The levels of blood ammonia before and after Rex shunt were normal, there was no patients suffering from encephalopathy.

## Discussion

Thrombosis of the bypass vein is considered to be an important cause of failure in the Rex shunt (11, 13, 14). Anticoagulant therapy is considered as an effective method to prevent postoperative venous thrombosis and treat portal vein thrombosis (5, 7, 24, 25). Especially for patients with abnormal coagulation mechanisms (such as a lack of anticoagulant substances), postoperative anticoagulant therapy is particularly important (26). However, the current anticoagulant regimens for Rex shunt are not uniform. The controversies of anticoagulant therapy for Rex shunt mainly include the start time using anticoagulant drugs (before, during, or after operation), the dosage of anticoagulant drugs, the choice of anticoagulant drugs, and the duration of anticoagulant therapy. The different centers performing Rex shunt have different anticoagulant strategies. The following postoperative anticoagulant strategies had been reported: 1. Administration of low dose (10 mg/kg/h) intravenous heparin for 3 days followed by oral aspirin and dipyridamole for 6 months (12). 2. Following surgery, children received unfractionated heparin (UFH) followed by dual antiplatelet agents (aspirin and dipyridamole) or received UFH followed by warfarin alone. Children received varying doses of UFH ranging from a prophylaxis regimen of 10 units/kg/h to therapeutic dosing that aimed to maintain APTT values within 60–85 s for 4 days after surgery. Postoperatively, patients were discharged on aspirin and dipyridamole orally for 6 months as primary prophylaxis for shunt thrombosis or on warfarin if considered to be at high risk for shunt thrombosis (7). Therefore, to identify the effectiveness of our postoperative anticoagulant strategy, this study was performed.

The selection of the bypass vein and the previous surgery history would affect the prognosis of the Rex shunt (3, 27). To avoid these influences, the patients who had no previous history of shunt surgery were included in this study, and the same method of Rex shunt (portal cavernoma- Rex shunt) was performed by the same surgeon.

It might seem counterintuitive to treat portal hypertension with anticoagulants for fear of increasing the frequency and/or intensity of future hemorrhage, which was also our cognition about using anticoagulant therapy after Rex shunt before 2018. To eliminate the fear, we need to identify the cause of hemorrhage in EHPVO. The rupture of esophageal and gastric varices caused by portal hypertension, instead of coagulation disorders, is the key reason for hemorrhage in EHPVO. Therefore, reducing portal pressure is an important method that decreasing the frequency and/or intensity of future hemorrhage. Keeping patency of the bypass vein after Rex shunt can help reduce portal pressure. We hypothesize that post-operative anticoagulant therapy plays a role in the reduction of re-bleeding by keeping the patency of the bypass vein and reducing portal pressure. This hypothesis is neither new nor untested. Kitchens CS et al. (24) found that chronic oral anticoagulant therapy is possibly effective in reducing portal hypertension in patients with hypercoagulability-induced extrahepatic portal hypertension.

This study found that the incidence of postoperative re-bleeding caused by varices in the non-anticoagulant group was higher than that in the anticoagulant group, and the patency rate of bypass vein in the non-anticoagulant group was lower than that in the anticoagulant group. These findings indicated that the anticoagulant therapy using heparin combined with Plavix is beneficial to maintain the patency of the bypass vein and reduce the risk of re-bleeding after Rex shunt. The patent bypass vein is the key to the success of the Rex shunt, however, which is not the only factor determining the surgical effectiveness. The surgical effectiveness is also affected by the volume of portal blood into the liver through the bypass vein, which might be more significant than the patency of the bypass vein, the diameter, and blood flow velocity of the bypass vein due to its role in evaluating the ability of the shunt. In this study, the diameter and flow velocity of the bypass vein measured by the US were used to calculate the flow volume (FV) into the liver through the bypass vein. The standard portal venous flow (SPFV) was used for elimination bias among different ages, which is the normal portal venous flow of children with a specified age. Through using SPFV, the bypass venous flow index (BVFI) was used to evaluate the ability of the Rex shunt that restore portal blood into the liver after eliminating bias among different ages. This study found that the BVFI of the anticoagulant group was significantly higher than that of the non-anticoagulant group. It suggested that heparin combined with Plavix can significantly improve the blood flow of the bypass vein into the liver, which may be the fundamental reason for a good prognosis after Rex shunt.

There are only 51 cases in this study due to the lower incidence of EHPVO in children, which inevitably affects the accuracy of results. In this study, there was no significant difference between the anticoagulant and non-anticoagulant groups in the remission rates of hypersplenism and esophagogastric varices due to the limited sample size. Therefore, to further evaluate the long-term efficacy of this anticoagulant regimen, it is still necessary to increase the number of cases in future studies to avoid bias caused by the smaller number of cases.

## Conclusion

In conclusion, heparin combined with Plavix can effectively reduce the risk of re-bleeding and maintain the patency of the bypass vein after Rex shunt, which may be working by improving the blood flow of the bypass vein into the liver.

## Data Availability

The raw data supporting the conclusions of this article will be made available by the authors, without undue reservation.
